# Mechanistic evaluation of primary human hepatocyte culture using global proteomic analysis reveals a selective dedifferentiation profile

**DOI:** 10.1007/s00204-016-1694-y

**Published:** 2016-04-02

**Authors:** James A. Heslop, Cliff Rowe, Joanne Walsh, Rowena Sison-Young, Roz Jenkins, Laleh Kamalian, Richard Kia, David Hay, Robert P. Jones, Hassan Z. Malik, Stephen Fenwick, Amy E. Chadwick, John Mills, Neil R. Kitteringham, Chris E. P. Goldring, B. Kevin Park

**Affiliations:** 1 Division of Molecular and Clinical Pharmacology, The Institute of Translational Medicine, MRC Centre for Drug Safety Science, The University of Liverpool, Liverpool, L69 3GE UK; 2 CN Bio, Centre for Innovation and Enterprise, Oxford University Begbroke Science Park, Begbroke, Oxfordshire OX5 1PF UK; 3 University Hospital Aintree, Longmoor Lane, Liverpool, L9 7AL UK; 4 AstraZeneca, Personalised Healthcare and Biomarkers, Alderley Park, Cheshire SK10 4TG UK

**Keywords:** Cytochrome P450s, Human hepatocytes, Mass spectrometry, Mitochondria, iTRAQ, Donor-variation

## Abstract

The application of primary human hepatocytes following isolation from human tissue is well accepted to be compromised by the process of dedifferentiation. This phenomenon reduces many unique hepatocyte functions, limiting their use in drug disposition and toxicity assessment. The aetiology of dedifferentiation has not been well defined, and further understanding of the process would allow the development of novel strategies for sustaining the hepatocyte phenotype in culture or for improving protocols for maturation of hepatocytes generated from stem cells. We have therefore carried out the first proteomic comparison of primary human hepatocyte differentiation. Cells were cultured for 0, 24, 72 and 168 h as a monolayer in order to permit unrestricted hepatocyte dedifferentiation, so as to reveal the causative signalling pathways and factors in this process, by pathway analysis. A total of 3430 proteins were identified with a false detection rate of <1 %, of which 1117 were quantified at every time point. Increasing numbers of significantly differentially expressed proteins compared with the freshly isolated cells were observed at 24 h (40 proteins), 72 h (118 proteins) and 168 h (272 proteins) (*p* < 0.05). In particular, cytochromes P450 and mitochondrial proteins underwent major changes, confirmed by functional studies and investigated by pathway analysis. We report the key factors and pathways which underlie the loss of hepatic phenotype in vitro, particularly those driving the large-scale and selective remodelling of the mitochondrial and metabolic proteomes. In summary, these findings expand the current understanding of dedifferentiation should facilitate further development of simple and complex hepatic culture systems.

**Electronic supplementary material:**

The online version of this article (doi:10.1007/s00204-016-1694-y) contains supplementary material, which is available to authorized users.

## Introduction

Primary human hepatocytes (PHHs) are an important tool for studying human liver disease and drug-induced liver injury (DILI), with other models, such as rat hepatocytes, hepatic cell lines and stem cell-derived hepatocyte-like cells failing to offer metabolically competent and species-relevant alternatives (Godoy et al. 2013). However, accessing and culturing PHHs remains difficult due to inconsistent supply and an inability to stimulate sufficient division for in vitro expansion. Furthermore, following isolation, primary human hepatocytes undergo a rapid dedifferentiation process in which many unique hepatocyte characteristics are lost, reducing the physiological relevance of the model (Fraczek et al. 2013; Godoy et al. 2013).

It is thought that dedifferentiation is caused by inflammatory and proliferative-associated responses to both PHH isolation and subsequent in vitro culture (Elaut et al. 2006; Fraczek et al. 2013; Godoy et al. 2009; Zellmer et al. 2010). Current models of dedifferentiation hypothesise that these responses result in the down-regulation of important liver-enriched transcription factors (LETFs) such as hepatic nuclear factors (HNFs), HNF1α and HNF4α, which control many of the ADME processes (absorption, distribution, metabolism and excretion) and other features unique to PHHs, resulting in a loss of hepatic phenotype (Godoy et al. 2009; Zellmer et al. 2010); however, the exact mechanisms which govern these changes remain incompletely understood.

Several approaches have been adopted to maintain the hepatocyte phenotype in culture, including co-culture and 3D models such as sandwich cultures, bioreactors, liver buds and spheroids which attempt to better replicate the in vivo hepatic environment (Darnell et al. 2012; No et al. 2012; Tostoes et al. 2012). In addition, a wide range of growth factors, cytokines and small molecules have also been used to target and maintain the hepatic phenotype, such as those shown recently by Shan et al. (Shan et al. 2013). Many of these strategies have been thoroughly reviewed by both Godoy et al. (2013) and Fraczek et al. (2013).

Despite these continued improvements and advances towards complex culture conditions which can maintain an improved phenotype over a period of weeks, the full in vivo phenotype has not yet been achieved. Therefore, there remains a real need to improve the understanding of the underlying causes of this process and to identify novel strategies/targets for intervention. Moreover, as complex models reduce the effects of dedifferentiation, analysis of these systems may result in the underlying causes being difficult to fully ascertain. Therefore, studies of more rudimentary models which result in more pronounced dedifferentiation may consequently provide a greater insight into the partially understood and currently unknown causes of dedifferentiation.

Crucially, increased understanding of dedifferentiation is also likely to be useful in guiding attempts to direct and maintain a hepatic phenotype from pluripotent cells in vitro. Current efforts worldwide have not taken a cell beyond a relatively immature hepatocyte (Kia et al. 2013), and it can be hypothesised that an integral part of this challenge is how we can capture the hepatic phenotype and prevent its loss.

Global studies offer an insight into how cells react in a holistic sense to a particular condition, and whilst efforts have been made to map changes in the hepatocyte transcriptome during dedifferentiation (Kim et al. 2010; Lasher et al. 2011), many changes key to the mature phenotype of the hepatocyte are not properly represented in such analysis. This is exemplified by the recent results, showing that only ~40 % of the variation in cell protein levels can be explained by differences in the corresponding mRNA levels (Schwanhausser et al. 2011). We have previously shown that the use of a global proteomic approach to phenotype rat hepatocytes can provide novel information on dedifferentiation (Rowe et al. 2010) and here look to build on that study using human cells.

We have therefore now followed PHHs over the first 7 days of monolayer culture, chosen specifically to exacerbate the effects of dedifferentiation. Using iTRAQ-based stable isotope labelling and pathway analysis, we have identified the key changes in the PHH proteome during dedifferentiation and the inter-donor variability, which exists not only in donor phenotype, but also in the subsequent dedifferentiation profile.

## Materials and methods

### Primary human hepatocyte isolation

Primary human hepatocytes were isolated using a previously described 2-step collagenase method (LeCluyse et al. 2005). Briefly, liver resections were received as surgical waste (Aintree hospital, Liverpool, UK) with full patient consent and ethical approval. The resections were perfused with HEPES-buffered saline (HBS; 10 mM Hepes, 5 mM KCl, 136 mM NaCl, 5 g/l glucose), followed by digestion with Collagenase A or IV (Roche, Basel, Switzerland or Sigma-Aldrich, St. Louis, MO) in HBS containing 700 µM CaCl_2_. The capsule was then opened and the digested cells separated using gauze. The suspension was centrifuged twice at 80xg for 5 min at 4 °C and resuspended in Williams E medium (Sigma-Aldrich, St. Louis, MO). Cells were plated onto collagen-I-coated plates (BD Dickinson, San Jose, CA) at 2.5 × 10^5^ cells/cm^2^ to achieve full confluency, in Williams E supplemented with 1 % insulin–transferrin–selenium (Life Technologies, Carlsbad, CA), 2 mM l-glutamine (Sigma-Aldrich, St. Louis, MO), 10^−7^ M dexamethasone and penicillin (100 units/ml)/streptomycin (100 µg/ml) (Sigma-Aldrich, St. Louis, MO). After 3 h, non-attached cells were washed away and the culture medium was replaced.

### iTRAQ

Fresh samples were collected directly after isolation, before cells were plated. Samples were spun at 80×*g* for 5 min and lysed in 100 µl iTRAQ buffer. In total, 24, 72 and 168 h time points were collected directly from 6 wells of a 24-well plates in a total of 100 µl iTRAQ buffer. Protein concentration was determined by Bradford assay. Protein lysates derived from five donors were labelled according to the manufacturer’s instructions (Applied Biosystems, Foster City, CA). One hundred micrograms protein in 20 µl of iTRAQ buffer was denatured, and the protein cysteine residues were reduced with tris(2-carboxyethyl)phosphine for 1 h at 60 °C and subsequently capped with methylmethanethiosulfate, before overnight digestion with reconstituted trypsin at 37 °C. Isopropanol was then added to each sample, before labelling with differentially weighted isobaric tags for 2 h, at room temperature. The labelled samples were then pooled and made up to 5 mL with 10 mM potassium dihydrogen phosphate/25 % w/v acetonitrile. The pH was then adjusted using concentrated phosphoric acid to <pH 3, before cation-exchange chromatography, followed by identification with mass spectrometry, as described previously (Rowe et al. 2010, 2013). Samples were run across three 8-plex iTRAQ runs (table S1), and results obtained relative to each donor’s fresh sample to control for inter-donor variation.

### Proteomic data analysis

Following iTRAQ analysis, only proteins, which were present in all samples, identified with 95 % confidence (2 or more peptides) or 99 % confidence (single peptide) with a false detection rate (FDR) of less than 1 % were statistically analysed using R open-source software (http://www.r-project.org/). The iTRAQ output was analysed and differentially expressed proteins (DEPs) identified using the linear models for microarray data (LIMMA) and *t* test (multtest) modules as described previously (Ritchie et al. 2015; Rowe et al. 2010). The R script and raw iTRAQ outputs used to generate the data are provided as supplementary information. Statistical outputs (*p* value, Benjamini–Hochberg and log fold change) of these modules were presented as volcano plots, and proteins detected in all samples were subjected to hierarchical clustering and heatmap analysis.

### Further proteomic data analysis

Individual trend analysis of CYPs and transporters detected in ≥3 donors was assessed by one-way ANOVA. Coefficient of variance (CV) was calculated as (standard deviation/mean). The most variable proteins were defined as CV > 1.3, and the most stable proteins were defined as CV < 0.3 and a mean relative fold change >0.8 and <1.25. PANTHER analysis was used to categorise differential subsets of proteins into biological functional groups and displayed as a pie chart (Mi et al. 2013).

### Pathway analysis

Significant DEPs (*p* < 0.05) were uploaded to the Ingenuity platform IPA (Qiagen, Venlo, Netherlands) to investigate the associated pathways, networks and regulators. IPA uses a database of known protein interactions from scientific publications to associate and group the uploaded DEPs with pathways (Thomas and Bonchev 2010). Using IPA algorithms, the software generates a *p* value which relates to the likelihood that a particular pathway or network is linked to the DEPs in the dataset. Only pathways that were altered by *p* < 0.05 (Fisher exact *t* test) were classed as significantly altered or linked. The Z-activation score, which additionally takes into account the directional change of the proteins, was used for biological function and upstream regulator analysis. Using the IPA algorithm, functions or regulators that have a Z-score of ≥2 are predicted to be activated and ≤−2 are predicted to be inhibited.

### Transcription factor binding analysis

Mapper_2_ online software was used to compare the predicted transcription factor binding sites (Marinescu et al. 2005). Analysis was completed using the collated database, analysing the sequence of each gene 2000 base pairs upstream of the transcription start site. The number of proteins of interest which interacted with each predicted transcription factor was then compared to determine the significance of each factor. Those factors which demonstrated enriched predicted binding within a subset of proteins (≥4 proteins) were classed of factors of interest.

### Western blotting

Samples collected in iTRAQ buffer were quantified by Bradford assay and assessed by western blot to validate iTRAQ results. Briefly, 5 µg samples were denatured at 80 °C in Laemmli sample buffer (Sigma-Aldrich, St. Louis, MO) and separated in 10 % polyacrylamide gels and then transferred to nitrocellulose membranes (G.E Healthcare, Buckinghamshire, UK). Following 1 h blocking in 10 % milk (Bio-Rad, Hercules, CA), primary antibodies directed against CYP2E1 (Abcam, Cambridge, UK; 1:5000), CYP2D6 (BD Gentest, San Jose, CA; 1:1000), CYP1A2 (Abcam, Cambridge, UK; 1:3000) were added overnight or for 15 min Actin (Abcam, Cambridge, UK; 1:10,000). Following washing, secondary mouse (1:10,000; CYP2D6, CYP1A2, Actin) or rabbit (1:5000; CYP2E1) antibodies were subsequently added for 1 h. Membranes were then washed and visualised using chemiluminescence.

### Metabolism studies

To determine the metabolic changes that occur in PHH during dedifferentiation, we used quantitative mass-spectrometry-based activity assays. PHHs were incubated for 15 min at 37 °C (5 % CO_2_) at a final substrate cocktail concentration of 1 mM testosterone (CYP3A4) and 0.25 mM dextromethorphan (CYP2D6) (both purchased from Sigma-Aldrich, St. Louis, MO) in MeOH or H_2_O, respectively (Fisher Scientific, Pittsburgh, PA), in culture media. 0.5 mM Phenacetin (Sigma-Aldrich, St. Louis, MO) in 100 % MeOH was then added to the incubation media (1:1 v/v) as a stop solution and an internal standard for LC–MS–MS analysis. The media containing the respective metabolites, 6β-OH-testosterone and dextrorphan, were then quantified by LC–MS-MS. This was repeated for the 24, 48, 72 and 168 h time points.

### Seahorse functional mitochondrial assay

PHH isolated from subsequent donors were cultured on collagen-I-coated (Life Technologies, Carlsbad, CA; 50 µg/ml in 0.02 M acetic acid), XF 96-well cell culture microplates (Seahorse biosciences, North Billerica, MA; 2.5 × 10^4^ cells/well). OXPHOS stress test medium was supplemented with 25 mM d-glucose, 2 mM l-glutamine and 1 mM sodium pyruvate (final concentration). The glycolytic stress test medium was prepared by adding 2 mM l-glutamine (final concentration). Both media were pre-warmed to 37 °C. PHH culture medium was removed and replaced with OXPHOS or glycolytic stress test medium. Cells were incubated in a CO_2_-free incubator at 37 °C for 1 h. 1 µM oligomycin, 0.25 µM FCCP and 1 µM rotenone–antimycin-A (OXPHOS stress test) and 25 mM glucose, 1 µM oligomycin, and 100 mM 2-deoxyglucose solutions (glycolytic stress test) were prepared. Prior to the rate measurements, the XFe96 Instrument (Seahorse biosciences, North Billerica, MA) allowed the oxygen partial pressure to reach equilibrium. The oxygen consumption rate (OCR) and extracellular acidification rate (ECAR) were measured simultaneously three times to establish a baseline rate. After each compound injection, conditions were allowed to return to normal oxygen tension and pH. The OCR and ECAR measurements for each well were recorded and reported as pmol/min and mpH/min, respectively, by XF Wave software. Results were displayed as a percentage of maximal OCR and ECAR or as relative fold change of each parameter between time points.

### Rotenone assay

PHH was plated at 1 × 10^5^ in 96-well collagen-I-coated plates. At 24 and 168 h, serial concentrations (0–20 mM) of rotenone (Sigma-Aldrich, St. Louis, MO) were made in DMSO (Fisher Scientific). Then, the compound solutions were added to culture media at 1/200 (v/v) ratio to make final dosing concentrations of 0–100 µM (0.5 % v/v DMSO). Culture media were removed and replaced with the media containing rotenone. Following incubation for 2 h (37 °C, 5 % CO_2_), ATP content was assessed using the CellTitre-Glo assay (Promega, Madison, WI), according to the manufacturers’ instructions. Briefly, 30 µl of ATP reagent was added to each well containing 100 µl of media. The plate was shaken (1 min), and then, 100 µl of the well content was transferred to a white 96-well plate, and the ATPase luminescence was measured using a plate reader (Varioskan, Thermo Scientific, Waltham, MA). Results were presented as percentage of control.

## Results

### Global proteomics reveals donor-dependent variation as a major influence on dedifferentiation

**Fig. 1:**
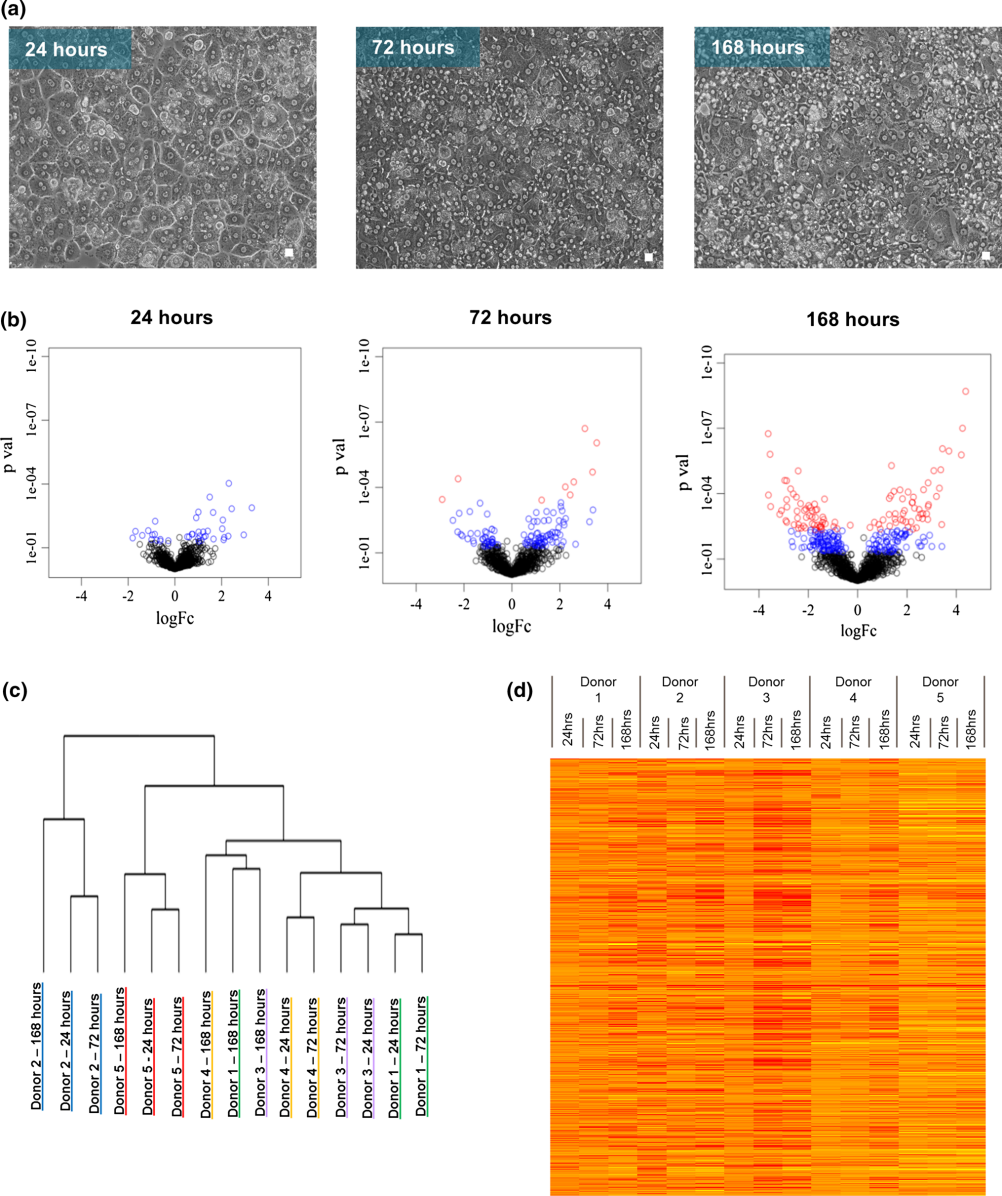
Morphological and statistical analysis **a** morphological changes in primary human hepatocytes over 168 h in monolayer collagen-I-coated plate culture. *Scale bar* represents 10 µm; **b** volcano plot analysis of the iTRAQ detected proteins log fold change versus *p* value at 24, 72 and 168 h. *Blue* significantly altered proteins (*n* = 5; *p* ≤ 0.05) and *red* significantly altered after multiple testing correction (*n* = 5; Benjamini–Hochberg ≤0.05) **c** hierarchal clustering analysis showing the relationship between donors and time points (*n* = 5); **d** heatmap of each donor’s proteome during dedifferentiation relative to the freshly isolated sample. *Red* indicates down-regulation, *yellow* up-regulation. The intensity of *colour* reflects the degree of change

iTRAQ quantitative global proteomic analysis was used to assess the changes which occur in PHH at different time points during a 168-h monolayer culture. Dedifferentiation and loss of characteristic hepatocyte morphology were confirmed by microscopy. Figure 1a shows images of the cells taken at 24, 72 and 168 h and clearly demonstrates the maintenance of confluency and the loss of the typical hepatocyte polygonal morphology (24 h), towards a culture with flatter and less defined epithelial characteristics at 72 and 168 h.

A total of 3430 unique proteins were detected across three iTRAQ runs (table S1), with 1117 identified across all samples. Statistical analysis using a linear model yielded significantly differentially expressed proteins (DEPs) at each time point relative to freshly isolated cells (table S2). These changes highlight a dynamic and escalating process; 40 DEPs are seen after 24 h, 118 after 72 h and 272 after 168 h in culture (*p* < 0.05). When displayed as volcano plots (log fold change vs. *p* value), the increasing dispersion and direction of change of the proteins over time in culture are strikingly evident (Fig. 1b); interestingly, at both 24 and 72 h of culture, the majority of DEPs increased in expression.

Hierarchical clustering of the entire dataset shows that the dominant factor in determining how the samples are related is in fact the original source of the cells, rather than time in culture (Fig. 1c). This is particularly pronounced at the 24 and 72 h time points, which cluster according to donor in every case. The separation from the other donors is still evident at 168 h for donors 2 and 5, although these samples do cluster away from the earlier time points, whereas by this time the other three donors (1, 3 and 4) cluster together, demonstrating a convergence towards a dedifferentiated phenotype which is more reflective of time in culture than the cell source. Thus, these data suggest that phenotypic characteristics of the donor have a greater impact on the protein dedifferentiating expression profile of the hepatocytes than does the length of time in culture, particularly over the first 72 h of culture. The variability between donor proteomic profiles during dedifferentiation is further shown by the heatmap in Fig. 1d.

To identify the proteins with the greatest variability across the 5 donors at the different time points, coefficient of variance (CV) analysis was then used (table S3). This criterion highlights a number of proteins, including CYP2C9, which is known to vary in expression between individuals (Sistonen et al. 2009), and glutamine synthetase, which is associated with differential expression according to liver zonation (Jungermann and Kietzmann 1996). In fact, zonation appears to be a large influencing factor on variation during culture as our pathway analysis of the most variable proteins revealed significant association with ketogenesis, cholesterol biosynthesis, fatty acid β oxidation and glutamine synthesis (table S4), all of these are related to the portocentral axis gradient of the lobule (Godoy et al. 2013).

The most stable proteins during dedifferentiation were also investigated (Table S5). These included several mitochondrial proteins within the ten most stable proteins. Thus, while many proteins show conserved differential expression or high levels of inter-donor variability, a subset of proteins remains relatively stable and consequently may retain in vivo-like functionality and also serve as useful normalisers for protein expression changes.

Table S6 highlights the most up- and down-regulated DEPs at each time point, as assessed by log_2_ fold change relative to the fresh sample. Interestingly, the first noticeable effects at 24 h are an increase in stress-related mediator superoxide dismutase and a decrease in histone proteins. Of note, hepatic-associated proteins are not universally down-regulated, as exemplified by alpha-1-antitrypsin and cytokeratins 8 and 18, which are up-regulated throughout the analysis (Table S2 and S6). Interestingly, these proteins are readily detectable in hepatocyte-like cells derived from stem cells (Rambhatla et al. 2003), whereas many of the down-regulated proteins are not, demonstrating that simple in vitro culture systems may support similar expression patterns during both hepatic dedifferentiation and differentiation.

### Pathway analysis reveals the key changes and dynamic profile of dedifferentiation

Pathway enrichment analysis was used to identify the affected functions and pathways which underlie dedifferentiation. A list of the most significantly perturbed pathways and functions at the three time points is shown in table S7.

**Fig. 2:**
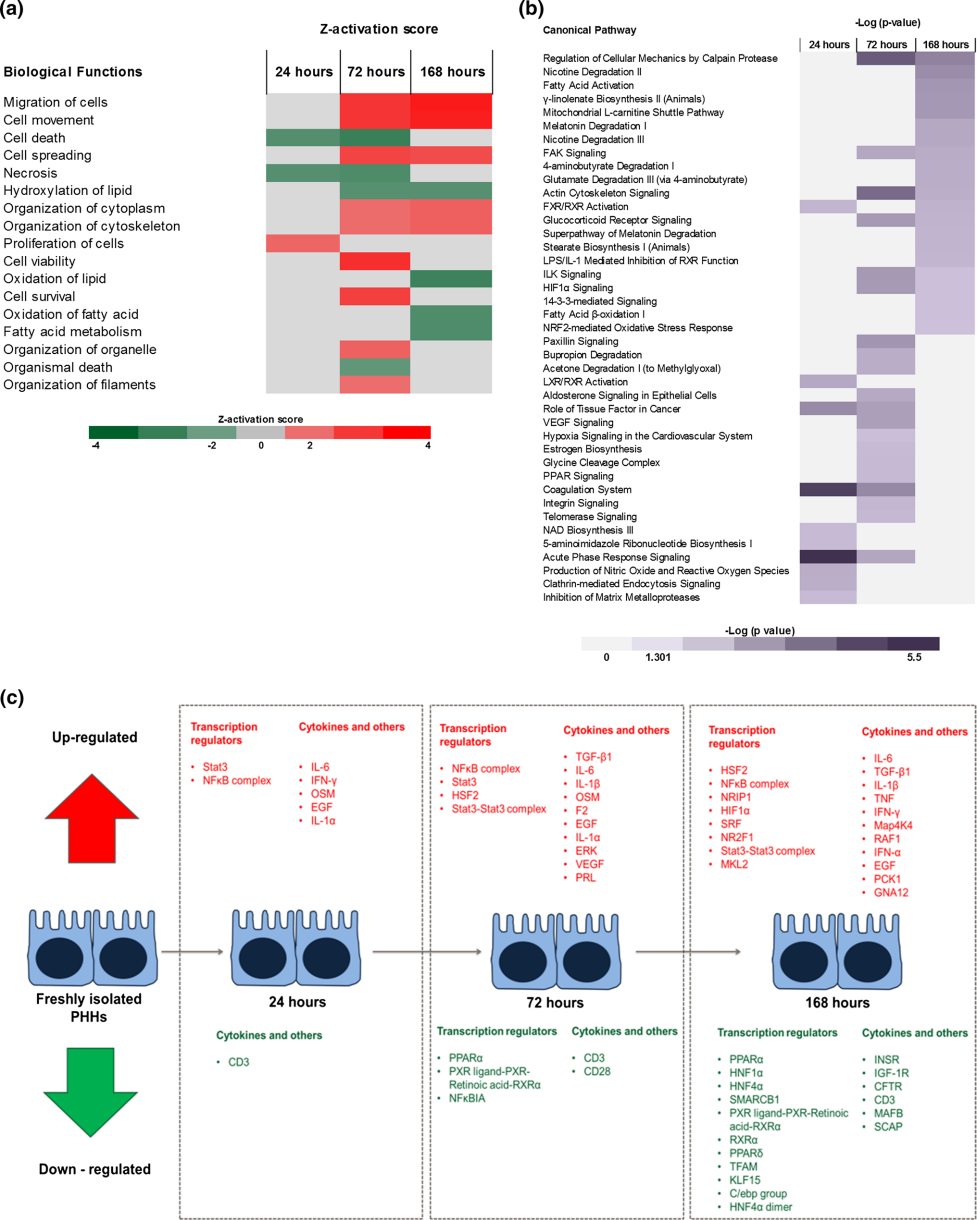
Pathway analysis of dedifferentiation **a** biological functions associated with the DEPs according to Z-activation score. If score 2 ≥ Z-score ≤ −2, the function is predicted to be activated or inhibited, respectively. *Red* up-regulated, *green* down-regulated. Intensity of colour correlates with greater Z-activation score **b** canonical pathways during dedifferentiation. Results displayed—Log (*p* values), pathway included if *p* < 0.05 at any of the assessed time points. *Colour* Intensity corresponds to the significance value; **c** upstream regulators predicted to be either activated or inhibited by Z-activation score (2 ≥ Z-score ≤ −2). *Red* activated, *green* inhibited, in order of significance

Heatmap analysis of the biological functions at the three post-isolation time points (Fig. 2a) shows the differential dynamics of the pathway groups. Many of the pathways follow a linear pattern of increasing activation or inhibition; however, some, such as the cell survival pathways, display different dynamics, as they are predicted to be activated at 72 h, but not at 24 or 168 h. Figure 2b displays the canonical pathways at the assessed time points. Whilst metabolic pathways display a trend of increasing significance during culture, acute phase response signalling and ROS/NOS production are significantly affected at 24 h, but not at 168 h. Taken together, these data indicate a time point-dependent cellular response to isolation and culture (figure S1).

Upstream regulation analysis (i.e. transcription factors, cytokines, growth factors and receptors) was then used to predict which regulators were activated or inhibited (Fig. 2c). The results suggest an activation of proliferative and inflammatory regulators and inhibition of metabolic-associated factors. Moreover, many energy production-associated factors are down-regulated at 168 h including PPARα, insulin receptor, KLF15 and the mitochondrial transcription factor TFAM.

### Analysis of mitochondrial functional profile reveals donor variation in the bioenergetic profile

**Fig. 3:**
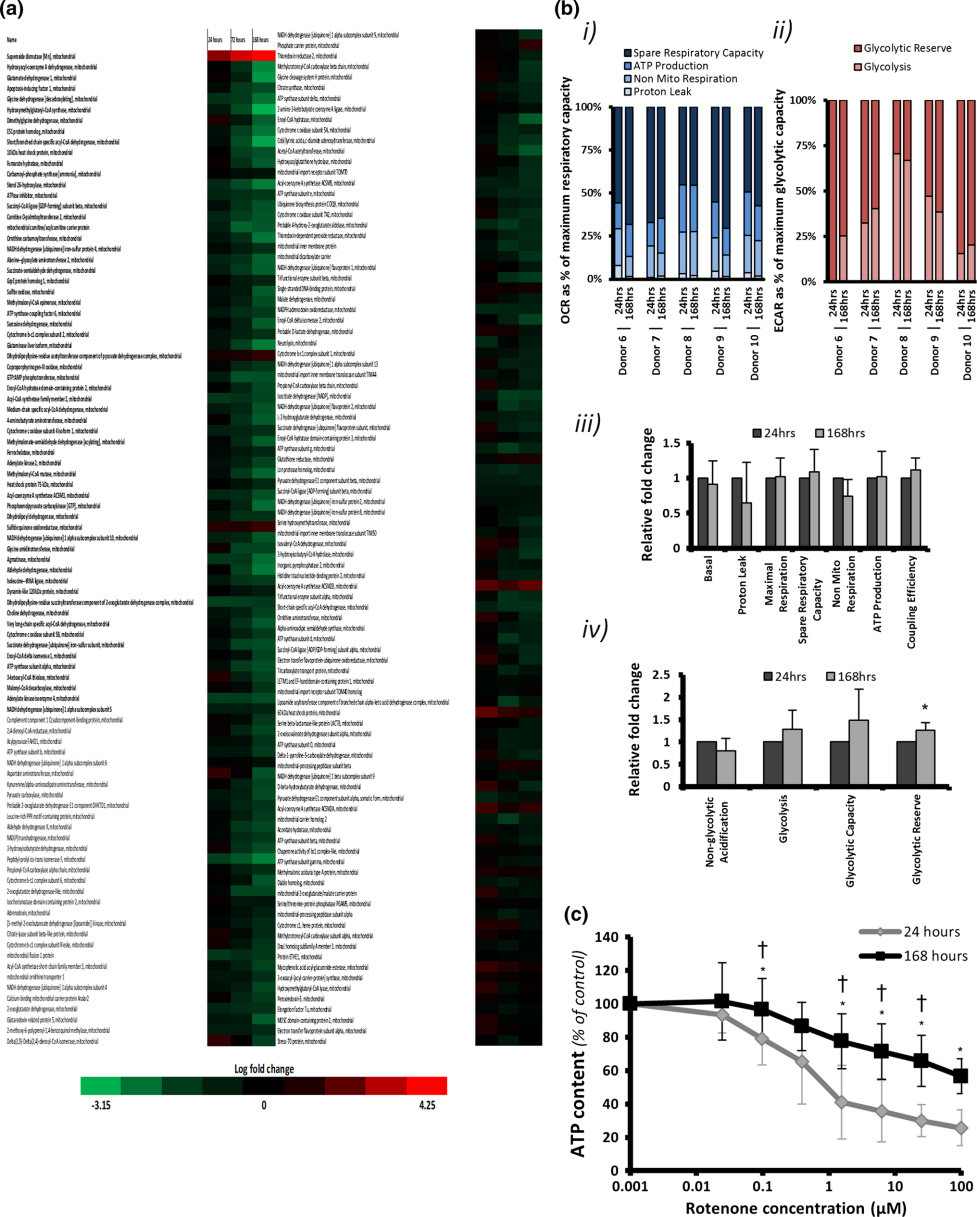
Mitochondrial changes during dedifferentiation. **a** Log_2_ fold change of mitochondrial proteins. *Red* up-regulated, *green* down-regulated. *Intensity of colour* log_2_ fold change. *Bold proteins* differentially expressed at 168 h (*p* < 0.05); **b** (*i*) oxidative phosphorylation and (*ii*) glycolysis parameters as percentages of maximal oxygen consumption rates and extracellular acidification rate, respectively; relative fold change of (*iii*) oxidative phosphorylation and (*iv*) glycolysis parameters. (*) *p* < 0.05 two-tailed paired t test; **c** ATP levels at 24 (*filled diamond*) and 168 h (*filled square*) following rotenone treatment. Results: percentage of control, (*) *p* < 0.05 two-tailed paired *t* test and (†) *p* < 0.05 Wilcoxon signed rank test (*n* = 5)

Pathway analysis predicted a perturbation of energy production and mitochondrial mechanics; therefore, we investigated the mitochondrial proteome in further detail. The expression of proteins with known mitochondrial associations demonstrated a general downward trend of the DEPs (bold) with time in culture, particularly at 168 h, with superoxide dismutase II being a notable exception (Fig. 3a). However, a number of key mitochondrial proteins, such as mitochondrial import receptor subunits, remained largely unchanged (table S5). This indicates that the large-scale down-regulation of mitochondrial proteins represents an alteration in function between freshly isolated cells and 168 h rather than a generalised loss of mitochondrial mass between these time points.

Pathway analysis was then used to assess the mitochondrial proteomic dataset DEPs. This analysis revealed predicted roles for AMPK signalling, a master regulator of energy metabolism, which increases in significance during time in culture (Figure S2a). Furthermore, a negative regulator of mitochondrial fusion, OMA-1, is also predicted to be down-regulated (figure S2b).

To assess how these changes impacted mitochondrial function, oxygen consumption rate (OCR) and extracellular acidification rate (ECAR) at 24 and 168 h were subsequently investigated in PHH isolated from a further five donors. The analysis demonstrated a clear variation in individual bioenergetic OCR profiles at 24 h (Fig. 3b (i)), with the 168-h profile showing greater dependence on the 24 h profile, rather than time in culture. Analysis of glycolytic consumption, as measured by ECAR, shows much greater donor-dependent variation (Fig. 3b (ii)), which may be attributable to the known gradient of glycolysis across the portocentral axis, and is consistent with our findings regarding the most variable proteins. Despite this variation, a trend emerges: basal glycolysis shows an increase during culture in donors with lower basal glycolysis, whereas those with higher basal levels at 24 h decrease, suggestive of a convergence towards a culture-dependent level of basal glycolysis.

Furthermore, many of the tested OCR parameters remain remarkably stable between 24 and 168 h (Fig. 3b (iii)), despite the large-scale changes to the mitochondrial proteome. ECAR measurements showed similar stability; however, there was a general trend of increased glycolytic parameters, including the only significantly altered factor, namely glycolytic reserve (Fig. 3b (iv)).

Further investigation of the proteomic data revealed a significant down-regulation of three complex I subunits, whereas the remaining nine detected subunits were not significantly differentially expressed. To assess the functional consequence of these changes, rotenone was used as a model of complex I mitochondrial perturbation. A significant increase in PHH resistance to rotenone toxicity in all donors at 168 h regardless of background bioenergetic (OCR/ECAR) profile (Fig. 3c) was found. Together these data suggest that, despite the variability seen between donors, a conserved change in essential mitochondrial function is observed as a result of time in culture.

### Assessment of ADME proteins during dedifferentiation reveals selective protein down-regulation

A well-known feature of hepatocyte dedifferentiation is the loss of the metabolic phenotype. To distinguish whether this is a selective or global loss of metabolic competence, several protein subsets generated in this analysis were compared: 168 h DEPs, 168 h non-DEPs, most stable proteins and the most variable proteins. PANTHER analysis was used to compare the associated biological function categories of each subset (Figure S3). This analysis revealed similar percentages of proteins in each class, with the greatest proportion of proteins associated with metabolism in all subsets, indicating a selective, rather than wholesale alteration, in the hepatocyte metabolic profile.

**Fig. 4:**
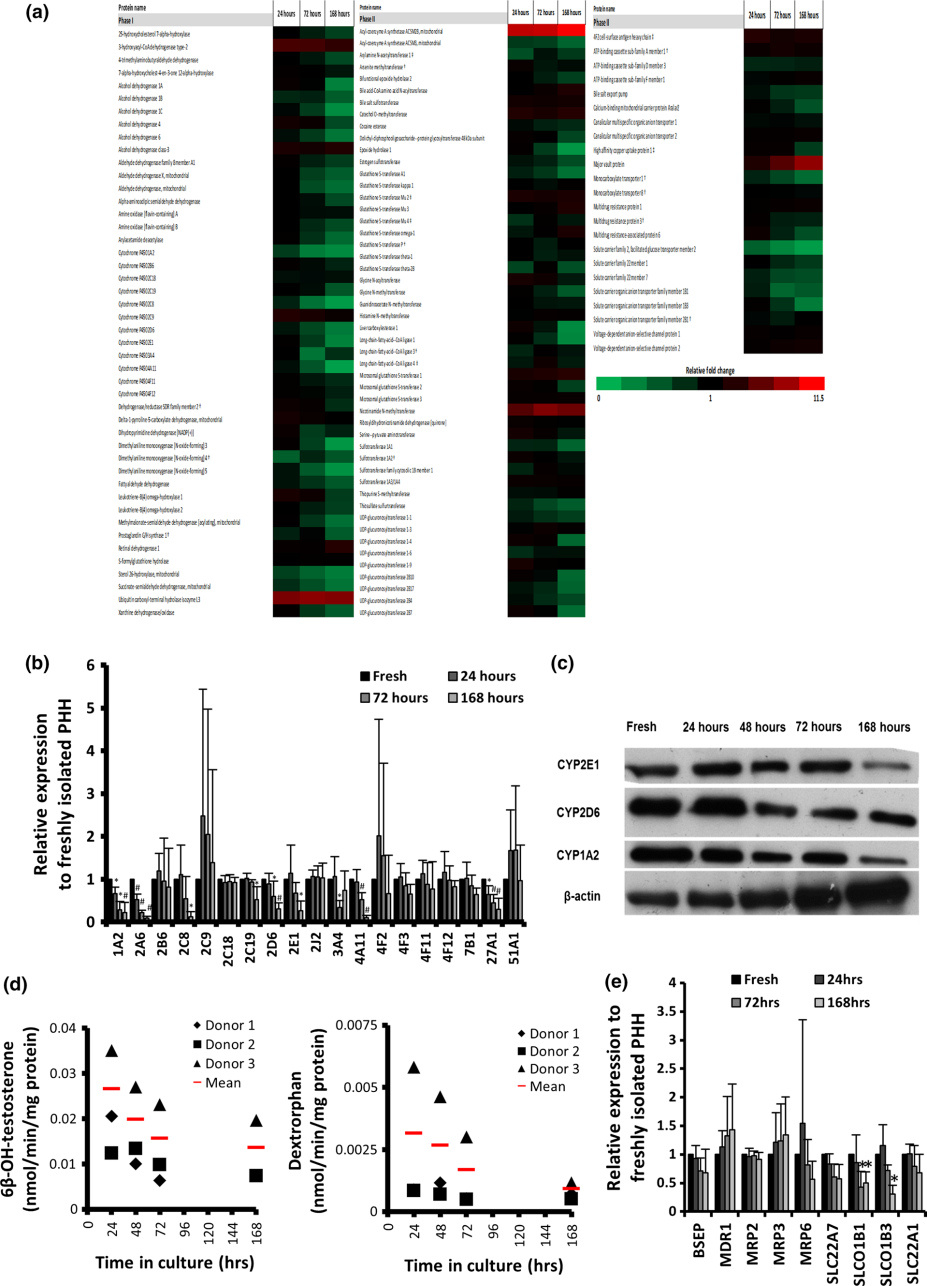
Loss of hepatic metabolic phenotype. **a** ADME phases I, II and III enzyme heatmap. *Red* up-regulated, *green* down-regulated. *Intensity of colour* relative fold change. Data derived from *n* ≥ 3 donors unless stated (†) *n* = 2 or (‡) *n* = 1; **b** CYP450 s over 168 h (*n* = 5); *error bars* SD; one-way ANOVA: (*) *p* < 0.05, (#) *p* < 0.01); **c** western blots for CYP450 s and β-actin; **d** metabolic function of CYP3A (testosterone) and CYP2D6 (dextromethorphan) and detection of respective metabolites (6β-OH-testosterone and dextrorphan) detected by LC–MS–MS (*n* = 3; *error bars* SD); **e** ADME transporters protein expression during dedifferentiation. *Error bars* SD,* significant *p* < 0.05 by one-way ANOVA

Figure 4a shows the known phase I, II and III drug-metabolising enzymes (Guo et al. 2011), which were detected by proteomic analysis. The heatmap highlights the loss of metabolic competence with the majority of phase I enzymes following a relatively linear pattern of down-regulation towards 168 h. Phase II/III enzymes appear to show greater stability with some phase III proteins, including multidrug resistance protein 1 (MDR1) and major vault protein (MVP), demonstrating an up-regulation during culture.

Figure 4b demonstrates the dedifferentiation pattern of the cytochrome P450 s detected by iTRAQ over the 168-h period. The general trend of expression relative to freshly isolated PHHs is downward; however, the majority of the detected CYP proteins are maintained in expression at 24 h in relation to the freshly isolated cells, showing that at 24 h the metabolic potential of PHH is mostly maintained (Fig. 4b and table S8). Some CYPs displayed greater stability, such as 2C18 and 4F11, whilst 2B6 and 2C9 demonstrated downward trends, but showed greater variation between the donors.

Western blotting for CYP proteins 2D6, 2E1 and 1A2 was consistent with the iTRAQ results, further demonstrating a downward trend with noted variation in the individual dedifferentiation profiles (Fig. 4c). Conversely, β-actin was up-regulated during the culture period, which was in keeping with the majority of cytoskeletal proteins detected by iTRAQ proteomics (n.b. iTRAQ relative quantification of β-actin is not included in table S2 as it did not meet the stringent criteria used for the analysis) and the pathway analysis (Fig. 2a). Furthermore, analysis of CYP450 activity using probe substrates for CYP3A and 2D6 shows a loss of functional capacity that is mostly consistent with the loss of protein recorded by iTRAQ and western immunoblotting (Fig. 4d). Interestingly, despite the increase in CYP3A4 protein at 168 h, no corresponding increase in CYP3A activity was detected; these results are in keeping with previous work which has shown that CYP protein expression does not correlate with activity in all situations (Rodriguez-Antona et al. 2002) and may represent the presence of the CYP3A4 protein in a non-functional state.

In contrast to the CYPs, the protein expression of the transporters identified as being important for drug influx and efflux in the liver (Zamek-Gliszczynski et al. 2012) is relatively stable throughout the 168-h culture. Only 2 of the 9 detected transporters are significantly down-regulated at 168 h (*p* < 0.05; Fig. 4e); interestingly, these are both influx transporters (SLCO1B1 and SLCO1B3). The general trend of the other detected drug influx transporters is also downwards, as are the trends for efflux transporters MRP6 and BSEP. Conversely, the remaining efflux transporters (MDR1, MRP2 and MRP3) show a maintenance or upward trend in expression during culture.

Comparison of the trend in the CYP and transporter proteins with previously published hepatocyte monolayer gene expression data (Richert et al. 2006) allows further investigation of the loss of metabolic competence (table S8). All CYPs with a reported gene expression >30 % at 72 h maintained protein expression >60 % at 168 h. Conversely, at 72 h, the mRNA of CYP1A2, 2A6, 2B6, 2C8, 2E1, 3A4, 4A11, 4F2, 4F3 and 27A1 is reported to drop to below 10 % of freshly isolated PHH (Richert et al. 2006). When the protein expression of these CYPs is compared, the majority, with the exception of CYP4F2 and 4F3, show significant down-regulation at 168 h, suggesting that the loss of CYPs can be mainly associated with reduced transcription. This association is shown in figure S4, demonstrating a good correlation between mRNA at 72 h and protein at 168 h in the down-regulated CYPs. Some phase III proteins appear to show greater stability, including BSEP, SLC22A7 and SLC22A1 which have mRNA <10 % at 72 h; however, despite a downward trend, they are not significantly down-regulated at 168 h.

### Pathway analysis of ADME proteins reveals the pathways and transcription factors predicted to underlie the loss of specific metabolic functions

Targeted analysis of the transcription factors that are associated with the ADME DEPs revealed the specific regulators, which are significantly perturbed and may cause the loss of ADME proteins during dedifferentiation (table S9). In keeping with published literature (Fraczek et al. 2013), HNF1α, C/EBP and HNF1β were amongst the most significantly associated regulators with the loss of metabolic competence. Interestingly, FECH (ferrochelatase), a key enzyme in the production of heme, found to be significantly down-regulated at 168 h (table S2), is highly associated with the differentially expressed ADME proteins. SMARCB1 is also significantly associated with the ADME proteomic profile at 168 h.

Investigation into the predicted transcription factor binding sites of differentially expressed and non-differentially expressed CYPs revealed the factors that may underlie the selectivity found in the loss of xenobiotic CYPs (Table S10). This analysis predicted binding sites for HSF2 to be highly enriched in the CYPs which were not differentially expressed, whereas ZEB1, FOXO1 and NF-Y binding sites are almost exclusively predicted to be located upstream of the differentially expressed CYPs.

## Discussion

In this study, we have demonstrated the first use of global proteomics as a tool for investigating primary human hepatocyte dedifferentiation, identifying new targets and generating new hypotheses for future examination in simple and complex culture models.

Throughout the analysis, differentially expressed proteins and associated pathways were found at every time point, suggesting that these mechanisms are well conserved across donors. The number of significantly differentially expressed proteins increased at each time, which, together with the clustering analysis, suggests an increasing deviation from the freshly isolated donor phenotype. Grouping of these data showed that dedifferentiation is not solely a change in expression of whole protein classes, but that it is a complex, differentially controlled and active process, in which selected pathways and functions from many classes are up- or down-regulated.

Cytochrome P450s has been strongly associated with a loss of expression during culture (Rodriguez-Antona et al. 2002), and this is further demonstrated here by the top three most down-regulated proteins at 168 h: CYP2A6, CYP2C8 and CYP4A11. However, this study allows for these changes to be put into the context of the global cell proteome, revealing the strikingly selective nature of dedifferentiation. Even within single P450 families, selectivity can be seen. For example, whilst CYP2A6 and CYP2C8 are the most down-regulated proteins, CYP2C18 and CYP2J2 remain unaltered by culture conditions. Of further interest, CYP2A6 has been reported to be the largest discriminant between foetal and adult hepatocytes (Rowe et al. 2013); here, we show CYP2A6 to be the largest discriminator between freshly isolated and 168 h cultured hepatocytes.

Understanding how this specificity works is a key question in determining new strategies for hepatocyte culture and for stem cell differentiation protocols. By comparing predicted transcription factor binding, we have been able to generate a list of enriched transcription factors associated with either differentially expressed or non-differentially expressed CYPs. Future investigations to elucidate the roles of these factors (e.g. HSF2, SP1 and ZEB1) may lead to a greater understanding of the selective loss/maintenance of metabolic competence during dedifferentiation. For example, ZEB1, a key mediator of epithelial–mesenchymal transition (EMT), may in part explain the link between the maintenance of epithelial phenotype and CYP expression.

Comparisons between differentially expressed and non-differentially expressed drug-metabolising proteins revealed no single factor which could mediate all the ADME-associated proteomic changes. The results corroborate previous reports showing the importance of HNF1α alongside HNF1β, C/EBP and FOXA1 in dedifferentiation (Padgham et al. 1993). Despite being a well-accepted major determinant of hepatic function (Odom et al. 2004), the lack of association of HNF4α with differentially expressed ADME proteins is consistent with HNF4α over-expression studies in rat hepatocytes, which failed to re-establish xenobiotic metabolic function (Naiki et al. 2005). The association of the enzyme ferrochelatase is also of interest. Ferrochelatase catalyses the final step of the heme production pathway, and thus, its loss of expression would likely reduce the intracellular heme concentration. CYPs are dependent on heme for function, and its loss during culture has been reported in mouse hepatocytes (Singh and Veltri 1991). HNF1α has been shown to regulate ferrochelatase expression (Muppala et al. 2000) and may provide an additional indirect mechanism by which HNF1α can alter ADME protein expression.

One potential cause of the change in the hepatocyte ADME and proteome-wide phenotype is through alterations in the accessibility of DNA to transcriptional machinery. Our results strongly predict a down-regulation of SMARCB1, a member of the BAF complex, which is thought to remodel chromatin structure (Wilson and Roberts 2011). SMARCB1 been shown to be essential for hepatocyte differentiation (Gresh et al. 2005), and recent work has also linked another member of the BAF complex, SMARCA4, to the enhancement of the hESC-derived HLC phenotype (Mobus et al. 2015). Alterations in chromatin accessibility and transcription factor binding are one of the key first steps in the reprogramming of somatic cells to induced pluripotent stem cells (Papp and Plath 2013), and similar fundamental mechanisms may play a role in the acquisition of the dedifferentiated phenotype. Efforts to intervene epigenetically using histone deacetylase and DNA methyltransferase inhibitors have been successful in maintaining expression of hepatic functions, although the exact mechanisms are unclear (Fraczek et al. 2012, 2013). Taken together, these results highlight the importance of epigenetic regulation in the dedifferentiation process; however, the consequences of these changes and the mechanisms which underlie them remain unclear and require further investigation.

Energy production is another fundamental cellular process affected by dedifferentiation that is highlighted by this study. The proteomic and functional data suggest a dramatic remodelling of the bioenergetic proteome during monolayer culture. These changes lead to a reduction in many mitochondrial-associated proteins, particularly those involved in lipid and fatty acid metabolism. Previous work has implicated AMPK signalling and mitochondrial fusion as part of the adaptation and repolarisation of hepatocytes in sandwich culture following isolation (Fu et al. 2013). Here we have additionally shown both AMPK and mitochondrial fusion pathways to be associated with the bioenergetic adaption to a monolayer culture system. Furthermore, given the remarkable stability of oxidative phosphorylation following plating, during a period of substantial phenotypic change, it appears that these essential processes are preferentially maintained, potentially at the expense of non-survival-essential proteins/functions. One possible driving factor of these findings is the MEK/ERK pathway, which is highlighted by this and other studies (Zellmer et al. 2010), as a factor in dedifferentiation and has previously been shown to reduce rotenone toxicity in neuronal cells (Jiang et al. 2006) and alter both mitochondrial function (Ripple et al. 2013) and lipid/fatty acid metabolism (Yousefi et al. 2012).

Our analysis revealed that the early stages of dedifferentiation are predominantly donor-dependent with the inter-individual variation that exists in the starting donor phenotype being maintained throughout the first 72 h of culture. Hepatocytes are known to vary between donors (due to both genotypic and environmental factors) (Bhogal et al. 2011) and in function depending on their location/zone in the lobule portocentral axis (Allen et al. 2005); both of these factors also appear to influence the dedifferentiation profile during culture. Similarity to the isolated donor phenotype dissipates with time in culture in all cases, a convergence towards a reproducible dedifferentiated phenotype was observed. Taken together, these data suggest that the strictly defined culture system promotes a specific protein expression profile, and, as a consequence of the variable PHH phenotype at isolation, the proteome changes required to achieve this dedifferentiated phenotype are also variable. This information has profound implications for the use of hepatocytes for early drug discovery and prediction of DILI; the majority of toxicological endpoints are assessed within 72 h of isolation; thus, the variable dedifferentiation of hepatocytes demonstrated in this study is likely to have an impact on attempts to achieve consistent biological assessment of multiple chemical variables (e.g. libraries of new drugs).

Many of the earliest changes in protein expression during dedifferentiation are related to cell survival and the maintenance of homoeostatic functions. The up-regulation of stress-induced survival pathways may be considered as a double-edged sword, on the one hand preventing apoptosis/necrosis and allowing cells to survive during culture, whilst on the other, playing an important role in the loss of the hepatic phenotype. Negative regulation of the hepatic phenotype has been reported to be true for the MEK/ERK and NFκB pathways (Elaut et al. 2006; Fraczek et al. 2013) and is predicted to be up-regulated by this study. Additionally, the most significantly predicted up-regulated factor at 168 h was heat shock factor 2 (HSF2). Overexpression studies have shown HSF2 to inhibit erythroid differentiation, but to our knowledge, HSF2 has not previously been linked to the hepatic differentiation status (Leppä et al. 1997). Such results emphasise the apparent trade-off between cell survival and a mature hepatic phenotype in traditional in vitro systems. Therefore, reducing cellular stress at all stages of culture, particularly during isolation, may be the most successful method of maintaining *both* cell viability and the hepatic phenotype.

Whilst some of the results yielded in this study may be specific to monolayer culture, the use of a rudimentary culture system has allowed the identification of a wide array of known and novel mechanisms through which dedifferentiation influences hepatic phenotype. We believe this approach enables a holistic understanding of the hepatocyte dedifferentiation process, and the knowledge gained in this study has the potential to enhance complex culture systems towards an improved physiological phenotype.

## Electronic supplementary material

Below is the link to the electronic supplementary material.

Supplementary material 1 (TIFF 796 kb)

Supplementary material 2 (TIFF 811 kb)

Supplementary material 3 (TIFF 860 kb)

Supplementary material 4 (TIFF 501 kb)

Supplementary material 5 (DOCX 94 kb)

Supplementary material 6 (XLSX 1131 kb)

Supplementary material 7 (XLSX 2868 kb)

Supplementary material 8 (TXT 9 kb)

Supplementary material 9 (CSV 597 kb)

Supplementary material 10 (CSV 356 kb)

Supplementary material 11 (CSV 730 kb)

Supplementary material 12 (CSV 732 kb)

Supplementary material 13 (CSV 597 kb)

Supplementary material 14 (DOCX 17 kb)

## Abbreviations

PHH: Primary human hepatocytes
DILI: Drug-induced liver injury
CYP: Cytochrome P450
LETFs: Liver-enriched transcription factors
HNF: Hepatic nuclear factor
HLC: Hepatocyte-like cell
MAPKs: Mitogen-activated protein kinases
NFκB: Nuclear factor kappa-light-chain-enhancer of activated B cells
DEPs: Differentially expressed proteins
ROS: Reactive oxygen species
HSF: Heat shock factor protein
ERK: Extracellular signal-regulated kinase
BSEP: Bile salt export pump
MDR: Multiple drug resistance protein
MRP: Multidrug resistance-related protein
SLC: Solute carrier family of transporters
