# Supplementary figures and images for: Mechanistic evaluation of primary human hepatocyte culture using global proteomic analysis reveals a selective dedifferentiation profile
Source: Arch Toxicol. 2016 Apr 2;91(1):439–52. doi: 10.1007/s00204-016-1694-y (PMC5225178; doi:10.1007/s00204-016-1694-y)

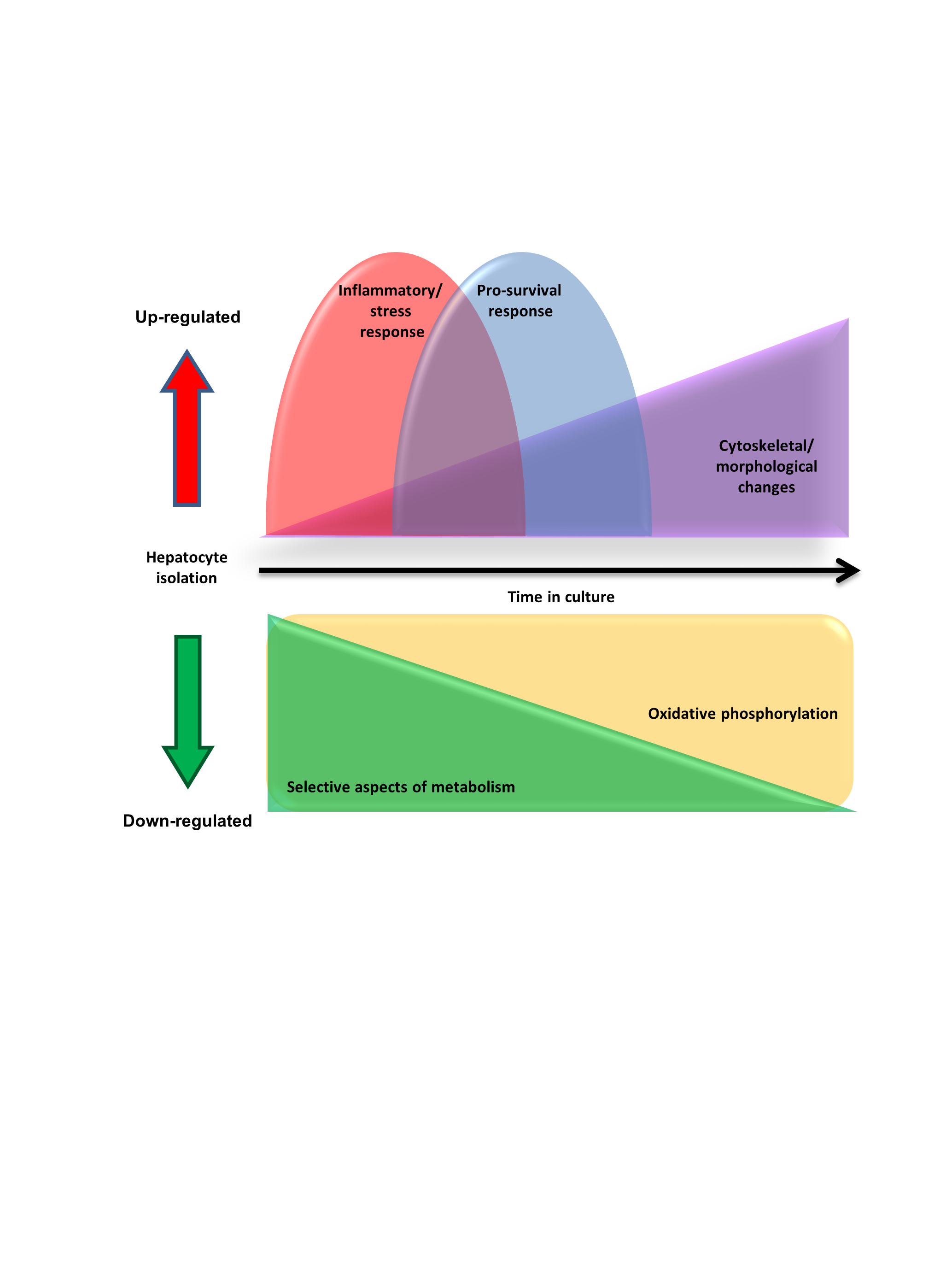

Supplement: Supplementary file 1 — Supplementary material 1 (TIFF 796 kb) [file 204_2016_1694_MOESM1_ESM.tif]

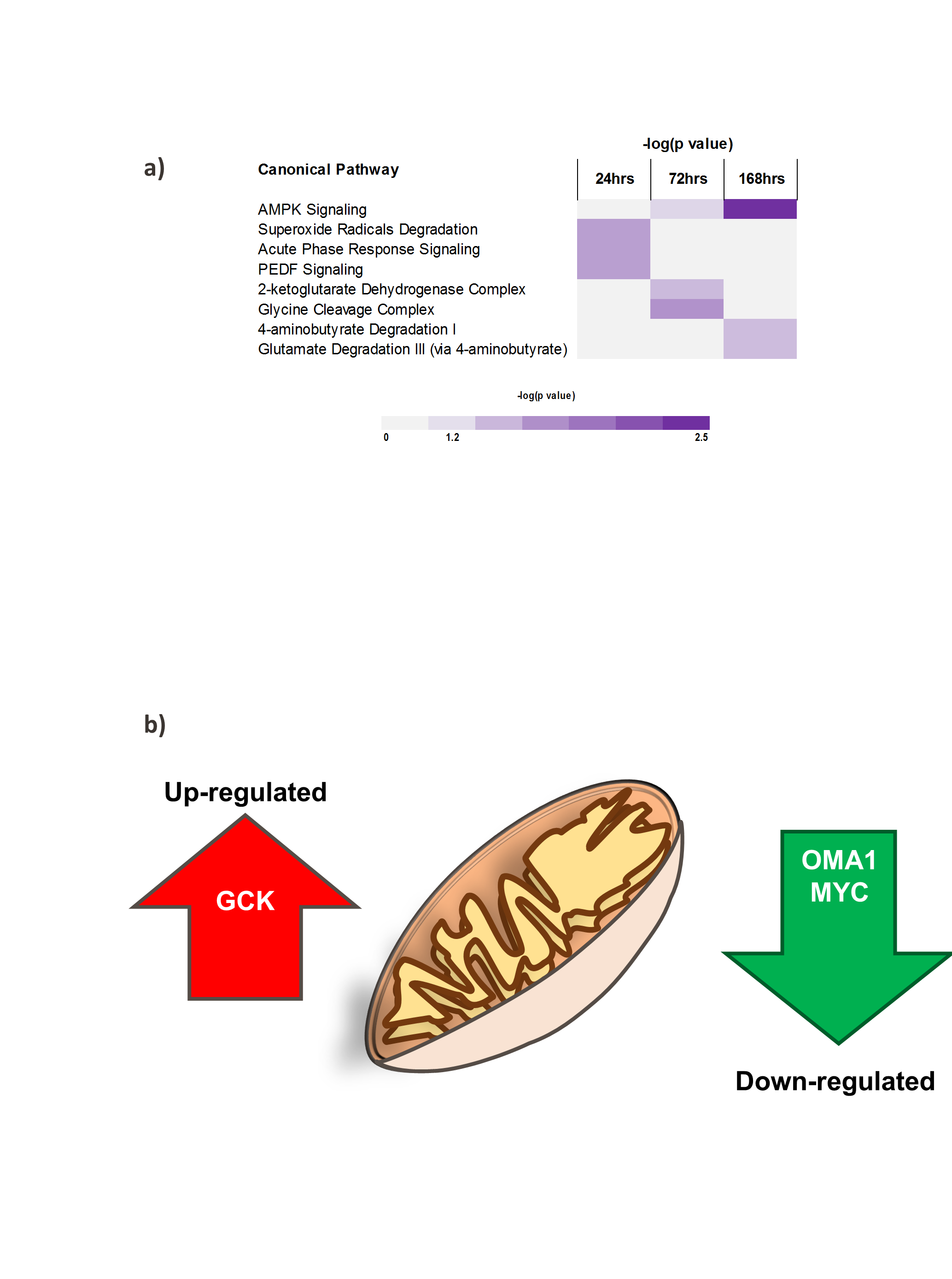

Supplement: Supplementary file 2 — Supplementary material 2 (TIFF 811 kb) [file 204_2016_1694_MOESM2_ESM.tif]

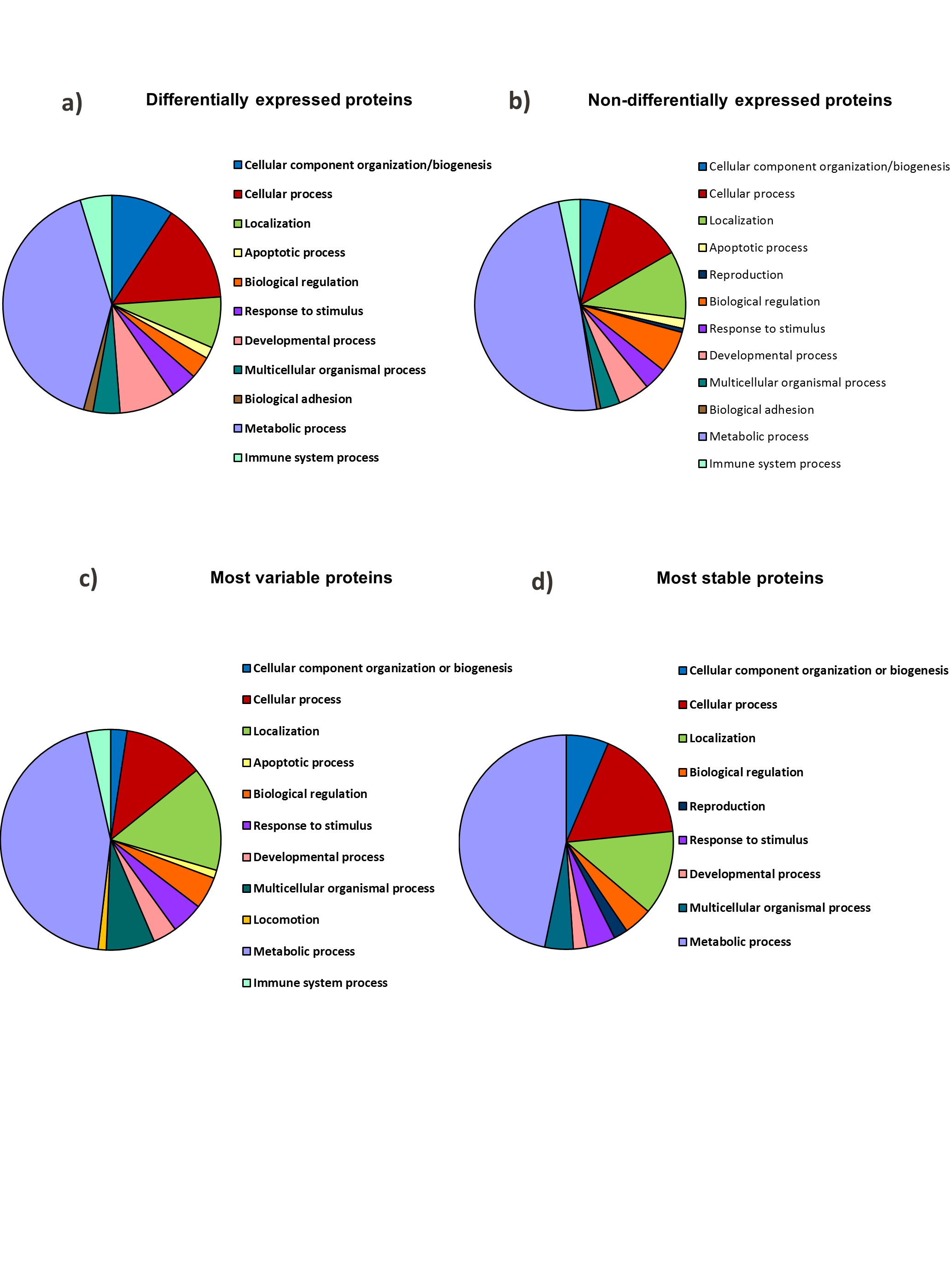

Supplement: Supplementary file 3 — Supplementary material 3 (TIFF 860 kb) [file 204_2016_1694_MOESM3_ESM.tif]

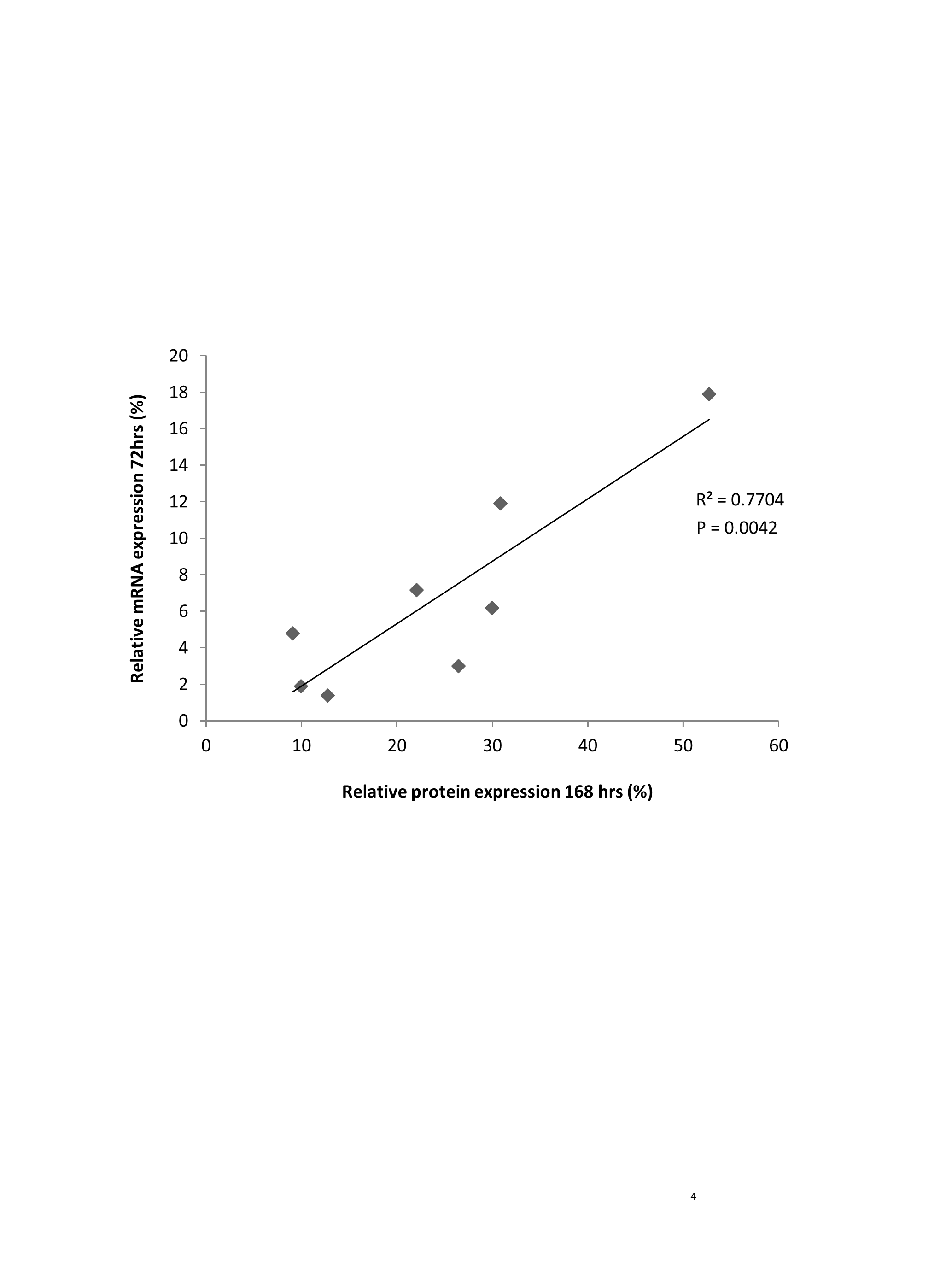

Supplement: Supplementary file 4 — Supplementary material 4 (TIFF 501 kb) [file 204_2016_1694_MOESM4_ESM.tif]
