# Supplementary material for: Mechanistic evaluation of primary human hepatocyte culture using global proteomic analysis reveals a selective dedifferentiation profile
Source: Arch Toxicol. 2016 Apr 2;91(1):439–52. doi: 10.1007/s00204-016-1694-y (PMC5225178; doi:10.1007/s00204-016-1694-y)
Supplement: Supplementary file 5 — Supplementary material 5 (DOCX 94 kb) [file 204_2016_1694_MOESM5_ESM.docx]

| Donors | Gender | Age | Reason for surgery | Underlying diseases | Medication | iTRAQ run | No. of proteins detected |
| --- | --- | --- | --- | --- | --- | --- | --- |
| Donor 1 | M | 67 | Colorectal liver metastases | Hypertension  Asthma  Hypercholesterolemia  Gout | Omeprazole  Atorvastatin  Tramadol  Allopurinol  Amlodipine  Lisinopril | 1 | 2439 |
| Donor 2 | M | 74 | Colorectal liver metastasis | Deep venous thrombosis  Benign prostatic hyperplasia | Citirizine  Finasteride  Terazosin  Paracetamol |  |  |
| Donor 3 | F | 61 | Cholangiocarcinoma | Severe pancreatitis | Diclofenac  Paracetamol  Omeprazole | 2 | 2944 |
| Donor 4 | M | 58 | Primary hepatocarcinoma | Hypertension | Atenadol  Allopurinol |  |  |
| Donor 5 | M | 66 | Colorectal liver metastases | Diabetes | Meiformin  Glicazide  Simvastatin | 3 | 2347 |

**Table S1**

**Table S2 – see Excel document**

| Uniprot Accession Number | Most variable proteins | *Co-efficient of variance* | | |
| --- | --- | --- | --- | --- |
|  |  | ***24 hours*** | ***72 hours*** | ***168 hours*** |
| P0DJI9 | Serum amyloid A-2 protein | 2.08 | 2.08 | 2.07 |
| Q9NUJ1 | Mycophenolic acid acyl-glucuronide esterase, mitochondrial | 1.71 | 1.88 | 1.99 |
| Q969Z3 | MOSC domain-containing protein 2, mitochondrial | 1.69 | 1.41 | 1.81 |
| P15374 | Ubiquitin carboxyl-terminal hydrolase isozyme L3 | 1.82 | 1.67 | 1.69 |
| P26599 | Polypyrimidine tract-binding protein 1 | 1.60 | 1.63 | 1.68 |
| O15260 | Surfeit locus protein 4 | 1.57 | 1.71 | 1.64 |
| O14756 | 17-beta-hydroxysteroid dehydrogenase type 6 | 1.09 | 1.35 | 1.64 |
| P25398 | 40S ribosomal protein S12 | 1.65 | 1.71 | 1.61 |
| P01011 | Alpha-1-antichymotrypsin | 0.70 | 0.42 | 1.58 |
| P11712 | Cytochrome P450 2C9 | 1.20 | 1.42 | 1.57 |
| Q96EK6 | Glucosamine 6-phosphate N-acetyltransferase | 1.74 | 1.61 | 1.54 |
| Q99714 | 3-hydroxyacyl-CoA dehydrogenase type-2 | 1.30 | 1.47 | 1.54 |
| Q96IJ6 | Mannose-1-phosphate guanyltransferase alpha | 1.67 | 1.35 | 1.51 |
| Q6YN16 | Hydroxysteroid dehydrogenase-like protein 2 | 1.47 | 1.60 | 1.50 |
| P24752 | Acetyl-CoA acetyltransferase, mitochondrial | 1.14 | 1.48 | 1.50 |
| Q16134 | Electron transfer flavoprotein-ubiquinone oxidoreductase, mitochondrial | 1.08 | 1.39 | 1.49 |
| P35914 | Hydroxymethylglutaryl-CoA lyase, mitochondrial | 1.13 | 1.40 | 1.48 |
| O43708 | Maleylacetoacetate isomerase | 1.40 | 1.52 | 1.48 |
| Q16222 | UDP-N-acetylhexosamine pyrophosphorylase | 1.58 | 1.63 | 1.47 |
| O75643 | U5 small nuclear ribonucleoprotein 200 kDa helicase | 1.44 | 1.33 | 1.44 |
| Q08AH3 | Acyl-coenzyme A synthetase ACSM2A, mitochondrial | 1.08 | 1.29 | 1.39 |
| P15104 | Glutamine synthetase | 0.35 | 1.05 | 1.39 |
| Q14558 | Phosphoribosyl pyrophosphate synthase-associated protein 1 | 1.17 | 1.09 | 1.38 |
| O75521 | Enoyl-CoA delta isomerase 2, mitochondrial | 1.33 | 1.49 | 1.38 |
| Q15366 | Poly(rC)-binding protein 2 | 1.70 | 1.41 | 1.38 |
| Q9BRX8 | Redox-regulatory protein FAM213A | 1.18 | 1.36 | 1.37 |
| P78329 | Leukotriene-B(4) omega-hydroxylase 1 | 1.35 | 1.40 | 1.37 |
| Q04446 | 1,4-alpha-glucan-branching enzyme | 1.15 | 1.04 | 1.36 |
| P62330 | ADP-ribosylation factor 6 | 1.47 | 1.57 | 1.33 |
| Q93088 | Betaine--homocysteine S-methyltransferase 1 | 0.85 | 0.88 | 1.32 |
| Q02338 | D-beta-hydroxybutyrate dehydrogenase, mitochondrial | 1.38 | 1.42 | 1.32 |
| Q68CK6 | Acyl-coenzyme A synthetase ACSM2B, mitochondrial | 1.39 | 1.37 | 1.32 |
| Q99624 | Sodium-coupled neutral amino acid transporter 3 | 1.36 | 1.16 | 1.32 |
| Q9BWD1 | Acetyl-CoA acetyltransferase, cytosolic | 1.58 | 1.45 | 1.29 |
| P30084 | Enoyl-CoA hydratase, mitochondrial | 1.00 | 0.99 | 1.29 |
| Q92820 | Gamma-glutamyl hydrolase | 1.22 | 1.47 | 1.28 |
| P05141 | ADP/ATP translocase 2 | 1.20 | 1.42 | 1.26 |
| P02042 | Hemoglobin subunit delta | 1.42 | 1.13 | 1.24 |
| Q02978 | Mitochondrial 2-oxoglutarate/malate carrier protein | 1.11 | 1.35 | 1.21 |
| P18085 | ADP-ribosylation factor 4 | 1.51 | 1.30 | 1.20 |
| Q3LXA3 | Bifunctional ATP-dependent dihydroxyacetone kinase/FAD-AMP lyase (cyclizing) | 1.40 | 1.22 | 1.20 |
| Q6NVY1 | 3-hydroxyisobutyryl-CoA hydrolase, mitochondrial | 1.38 | 1.65 | 1.18 |
| O95394 | Phosphoacetylglucosamine mutase | 1.37 | 1.31 | 1.16 |
| P09417 | Dihydropteridine reductase | 1.48 | 1.20 | 1.16 |
| P37837 | Transaldolase | 1.52 | 1.52 | 1.15 |
| Q96LJ7 | Dehydrogenase/reductase SDR family member 1 | 1.38 | 1.47 | 1.15 |
| P16219 | Short-chain specific acyl-CoA dehydrogenase, mitochondrial | 1.45 | 1.14 | 1.11 |
| P07339 | Cathepsin D | 1.30 | 1.51 | 1.08 |
| P07437 | Tubulin beta chain | 1.61 | 1.04 | 0.98 |
| P11766 | Alcohol dehydrogenase class-3 | 1.32 | 1.12 | 0.92 |
| P02753 | Retinol-binding protein 4 | 1.08 | 1.34 | 0.85 |
| Q8TC12 | Retinol dehydrogenase 11 | 1.45 | 0.87 | 0.84 |
| Q13885 | Tubulin beta-2A chain | 1.55 | 1.06 | 0.82 |

**Table S4**

| **Canonical Pathways** | **-log(p-value)** |
| --- | --- |
| Ketogenesis | 8.15 |
| Isoleucine Degradation I | 7.48 |
| UDP-N-acetyl-D-glucosamine Biosynthesis II | 6.54 |
| Ketolysis | 5.92 |
| UDP-N-acetyl-D-galactosamine Biosynthesis II | 5.92 |
| Glutaryl-CoA Degradation | 5.5 |
| Tryptophan Degradation III (Eukaryotic) | 4.73 |
| Fatty Acid β-oxidation I | 4.21 |
| Mevalonate Pathway I | 3.33 |
| Remodeling of Epithelial Adherens Junctions | 3.19 |
| Superpathway of Geranylgeranyldiphosphate Biosynthesis I (via Mevalonate) | 3.09 |
| Valine Degradation I | 3.04 |
| Superpathway of Cholesterol Biosynthesis | 2.66 |
| Glutathione-mediated Detoxification | 2.63 |
| Glutamine Biosynthesis I | 2.6 |
| Glutamate Removal from Folates | 2.6 |
| Ethanol Degradation II | 2.52 |
| Noradrenaline and Adrenaline Degradation | 2.47 |
| Estrogen Biosynthesis | 2.4 |
| Formaldehyde Oxidation II (Glutathione-dependent) | 2.3 |

|  |  | **Mean fold change** | | | **Co-efficient of variance** | | |
| --- | --- | --- | --- | --- | --- | --- | --- |
| **Accession** | **Most stable proteins** | **24 hours** | **72 hours** | **168 hours** | **24 hours** | **72 hours** | **168 hours** |
| O75600 | 2-amino-3-ketobutyrate coenzyme A ligase, mitochondrial | 0.99 | 1.00 | 0.86 | 0.10 | 0.29 | 0.07 |
| **P31327** | **Carbamoyl-phosphate synthase [ammonia], mitochondrial** | **0.98** | **0.87** | **0.81** | **0.04** | **0.11** | **0.08** |
| Q5SRE7 | Phytanoyl-CoA dioxygenase domain-containing protein 1 | 1.01 | 0.99 | 0.94 | 0.20 | 0.17 | 0.08 |
| O95202 | LETM1 and EF-hand domain-containing protein 1, mitochondrial | 0.96 | 0.92 | 0.93 | 0.25 | 0.09 | 0.08 |
| O14818 | Proteasome subunit alpha type-7 | 1.02 | 1.10 | 1.12 | 0.15 | 0.13 | 0.09 |
| **P35606** | **Coatomer subunit beta'** | **1.15** | **1.13** | **1.19** | **0.08** | **0.08** | **0.09** |
| Q16531 | DNA damage-binding protein 1 | 0.90 | 0.87 | 0.98 | 0.12 | 0.15 | 0.10 |
| Q07954 | Prolow-density lipoprotein receptor-related protein 1 | 0.83 | 0.89 | 0.98 | 0.14 | 0.15 | 0.11 |
| P55735 | Protein SEC13 homolog | 1.12 | 1.02 | 1.24 | 0.10 | 0.23 | 0.11 |
| O96008 | Mitochondrial import receptor subunit TOM40 homolog | 0.98 | 0.93 | 1.10 | 0.20 | 0.28 | 0.13 |
| P62249 | 40S ribosomal protein S16 | 0.98 | 1.13 | 1.15 | 0.29 | 0.22 | 0.14 |
| P56556 | NADH dehydrogenase [ubiquinone] 1 alpha subcomplex subunit 6 | 0.98 | 0.97 | 0.84 | 0.10 | 0.14 | 0.14 |
| P62847 | 40S ribosomal protein S24 | 1.15 | 1.19 | 1.09 | 0.27 | 0.13 | 0.15 |
| P30533 | Alpha-2-macroglobulin receptor-associated protein | 1.01 | 0.94 | 0.94 | 0.09 | 0.10 | 0.16 |
| Q13586 | Stromal interaction molecule 1 | 0.95 | 1.01 | 0.84 | 0.09 | 0.14 | 0.17 |
| Q13131 | 5'-AMP-activated protein kinase catalytic subunit alpha-1 | 1.00 | 0.94 | 1.03 | 0.16 | 0.13 | 0.17 |
| Q8NC51 | Plasminogen activator inhibitor 1 RNA-binding protein | 1.06 | 0.97 | 0.95 | 0.19 | 0.17 | 0.17 |
| Q8NI60 | Chaperone activity of bc1 complex-like, mitochondrial | 0.99 | 1.13 | 0.94 | 0.26 | 0.29 | 0.17 |
| Q16706 | Alpha-mannosidase 2 | 0.95 | 1.18 | 1.01 | 0.26 | 0.28 | 0.17 |
| O75131 | Copine-3 | 0.99 | 0.91 | 0.91 | 0.10 | 0.16 | 0.18 |
| Q86UE4 | Protein LYRIC | 1.05 | 0.96 | 1.04 | 0.23 | 0.12 | 0.18 |
| Q9H0R4 | Haloacid dehalogenase-like hydrolase domain-containing protein 2 | 1.13 | 1.13 | 1.19 | 0.17 | 0.06 | 0.19 |
| A1L0T0 | Acetolactate synthase-like protein | 0.93 | 0.90 | 0.83 | 0.11 | 0.26 | 0.19 |
| Q9H0E2 | Toll-interacting protein | 0.88 | 1.09 | 1.16 | 0.17 | 0.18 | 0.19 |
| P35637 | RNA-binding protein FUS | 0.92 | 1.00 | 1.02 | 0.18 | 0.14 | 0.19 |
| P51580 | Thiopurine S-methyltransferase | 0.93 | 0.94 | 1.16 | 0.30 | 0.25 | 0.19 |
| Q96HS1 | Serine/threonine-protein phosphatase PGAM5, mitochondrial | 1.11 | 0.99 | 0.98 | 0.20 | 0.28 | 0.20 |
| P10155 | 60 kDa SS-A/Ro ribonucleoprotein | 0.96 | 0.97 | 1.14 | 0.11 | 0.10 | 0.21 |
| O95340 | Bifunctional 3'-phosphoadenosine 5'-phosphosulfate synthase 2 | 0.91 | 1.11 | 1.13 | 0.23 | 0.15 | 0.21 |
| Q9Y6B6 | GTP-binding protein SAR1b | 0.95 | 0.94 | 0.91 | 0.30 | 0.24 | 0.21 |
| O14841 | 5-oxoprolinase | 1.00 | 0.98 | 1.01 | 0.08 | 0.22 | 0.22 |
| P23246 | Splicing factor, proline- and glutamine-rich | 1.08 | 1.05 | 1.09 | 0.22 | 0.24 | 0.23 |
| O95486 | Protein transport protein Sec24A | 1.06 | 0.94 | 1.15 | 0.16 | 0.27 | 0.23 |
| O75531 | Barrier-to-autointegration factor | 1.12 | 0.88 | 1.24 | 0.23 | 0.14 | 0.25 |
| Q969X5 | Endoplasmic reticulum-Golgi intermediate compartment protein 1 | 1.21 | 1.14 | 1.23 | 0.21 | 0.21 | 0.25 |
| Q9Y2T3  **Table S5** | Guanine deaminase | 1.19 | 1.03 | 1.25 | 0.24 | 0.27 | 0.27 |
| O43390 | Heterogeneous nuclear ribonucleoprotein R | 0.81 | 0.91 | 0.83 | 0.26 | 0.19 | 0.27 |
| P14868 | Aspartate--tRNA ligase, cytoplasmic | 0.91 | 1.13 | 0.98 | 0.26 | 0.15 | 0.30 |
| P62753  **Table S5** | 40S ribosomal protein S6 | 1.02 | 1.00 | 1.04 | 0.13 | 0.17 | 0.30 |

**Table S5**

| 24 hours | | | | | | 72 hours | | | | | | 168 hours | | | | | |
| --- | --- | --- | --- | --- | --- | --- | --- | --- | --- | --- | --- | --- | --- | --- | --- | --- | --- |
| Up-regulated | | | **Down-regulated** | | | **Up-regulated** | | | **Down-regulated** | | | **Up-regulated** | | | **Down-regulated** | | |
| Uniprot Accession Number | **Name** | **Log_2_ fold change** | **Uniprot Accession Number** | **Name** | **Log_2_ fold change** | **Uniprot Accession Number** | **Name** | **Log_2_ fold change** | **Uniprot Accession Number** | **Name** | **Log_2_ fold change** | **Uniprot Accession Number** | **Name** | **Log_2_ fold change** | **Uniprot Accession Number** | **Name** | **Log_2_ fold change** |
| P01011 | Alpha-1-antichymotrypsin | 3.293 | P62805 | Histone H4 | -1.809 | P04179 | Superoxide dismutase [Mn], mitochondrial | 3.54 | Q71DI3 | Histone H3.2 | -2.914 | P07355 | Annexin A2 | 4.377 | P11509 | Cytochrome P450 2A6 | -3.628 |
| P01009 | Alpha-1-antitrypsin | 2.947 | Q71DI3 | Histone H3.2 | -1.689 | P01011 | Alpha-1-antichymotrypsin | 3.383 | O14832 | Phytanoyl-CoA dioxygenase, peroxisomal | -2.465 | P04179 | Superoxide dismutase [Mn], mitochondrial | 4.249 | P10632 | Cytochrome P450 2C8 | -3.616 |
| P02679 | Fibrinogen gamma chain | 2.432 | P10412 | Histone H1.4 | -1.395 | P10909 | Clusterin | 3.367 | P62805 | Histone H4 | -2.27 | P10909 | Clusterin | 4.204 | Q02928 | Cytochrome P450 4A11 | -3.551 |
| P04179 | Superoxide dismutase [Mn], mitochondrial | 2.307 | P36957 | Dihydrolipoyllysine-residue succinyltransferase component of 2-oxoglutarate dehydrogenase complex, mitochondrial | -1.140 | P43490 | Nicotinamide phosphoribosyltransferase | 3.24 | P11509 | Cytochrome P450 2A6 | -2.248 | P37802 | Transgelin-2 | 3.699 | P11168 | Solute carrier family 2, facilitated glucose transporter member 2 | -3.528 |
| P43490 | Nicotinamide phosphoribosyltransferase | 2.283 | Q96CM8 | Acyl-CoA synthetase family member 2, mitochondrial | -1.001 | P07355 | Annexin A2 | 3.046 | P10412 | Histone H1.4 | -2.204 | P35579 | Myosin-9 | 3.439 | P54868 | Hydroxymethylglutaryl-CoA synthase, mitochondrial | -3.159 |
| P02671 | Fibrinogen alpha chain | 2.115 | P11509 | Cytochrome P450 2A6 | -0.959 | P01009 | Alpha-1-antitrypsin | 2.654 | P11168 | Solute carrier family 2, facilitated glucose transporter member 2 | -2.137 | P60903 | Protein S100-A10 | 3.412 | P31513 | Dimethylaniline monooxygenase [N-oxide-forming] 3 | -3.088 |
| P02763 | Alpha-1-acid glycoprotein 1 | 2.086 | P05386 | 60S acidic ribosomal protein P1 | -0.866 | P37802 | Transgelin-2 | 2.583 | P05177 | Cytochrome P450 1A2 | -2.076 | P43490 | Nicotinamide phosphoribosyltransferase | 3.407 | P07099 | Epoxide hydrolase 1 | -2.955 |
| P02675 | Fibrinogen beta chain | 2.054 | P46777 | 60S ribosomal protein L5 | -0.845 | P26038 | Moesin | 2.431 | P22307 | Non-specific lipid-transfer protein | -1.851 | P26038 | Moesin | 3.370 | P45954 | Short/branched chain specific acyl-CoA dehydrogenase, mitochondrial | -2.911 |
| P08107 | Heat shock 70 kDa protein 1A/1B | 2.028 | P14920 | D-amino-acid oxidase | -0.776 | P02679 | Fibrinogen gamma chain | 2.243 | O43772 | Mitochondrial carnitine/acylcarnitine carrier protein | -1.760 | P21333 | Filamin-A | 3.314 | P49326 | Dimethylaniline monooxygenase [N-oxide-forming] 5 | -2.880 |
| P07602 | Proactivator polypeptide | 1.653 | P62750 | 60S ribosomal protein L23a | -0.625 | P35579 | Myosin-9 | 2.23 | P08684 | Cytochrome P450 3A4 | -1.724 | P09493 | Tropomyosin alpha-1 chain | 3.194 | P04040 | Catalase | -2.879 |

**Table S6**

| **24 hours** |  | **72 hours** |  | **168 hours** |
| --- | --- | --- | --- | --- |
| **Cellular and molecular pathways** |  |  |  |  |
| - **Cellular Movement** - **Cell-To-Cell Signalling and Interaction** - **Cellular Growth and Proliferation** - **Cellular Development** - **Cellular Assembly and Organization** |  | - **Cell Death and Survival** - **Cellular Movement** - **Cellular Assembly and Organization** - **Cellular Function and Maintenance** - **Cell Morphology** |  | - **Energy Production** - **Lipid Metabolism** - **Small Molecule Biochemistry** - **Cellular Function and Maintenance** - **Cell Morphology** |
| **Toxicity pathways** |  |  |  |  |
| - **Positive Acute Phase Response Proteins** - **LXR/RXR Activation** |  | - **Positive Acute Phase Response Proteins** - **NRF2-mediated Oxidative Stress Response** - **CYP450 Panel - Substrate is a Xenobiotic (Human)** - **Aryl Hydrocarbon Receptor Signaling** - **Hypoxia-Inducible Factor Signalling** |  | - **NRF2 oxidative stress response** - **Fatty acid metabolism** - **LPS/IL-1 mediated inhibition of RXR function** - **Xenobiotic metabolism signalling** - **Positive acute phase response proteins** - **CYP450 panel – substrate is Xenobiotic (Human)** |
| **Networks** |  |  |  |  |
| - **Developmental Disorder, Hematological Disease, Hereditary Disorder** - **Lipid Metabolism, Small Molecule Biochemistry, Molecular Transport** - **Cellular Assembly and Organization, Amino Acid Metabolism, Small Molecule Biochemistry** |  | - **Post-Translational Modification, Protein Folding, Drug Metabolism** - **Cellular Assembly and Organization, Cellular Function and Maintenance, Cell Morphology** - **Protein Synthesis, Cell Death and Survival, Cellular Assembly and Organization** - **Drug Metabolism, Developmental Disorder, Hematological Disease** - **Nucleic Acid Metabolism, Small Molecule Biochemistry, Drug Metabolism** |  | - **Cell Death and Survival, Neurological Disease, Cancer** - **Small Molecule Biochemistry, Drug Metabolism, Nucleic Acid Metabolism** - **Lipid Metabolism, Molecular Transport, Small Molecule Biochemistry** - **Lipid Metabolism, Small Molecule Biochemistry, Vitamin and Mineral Metabolism** - **Cellular Assembly and Organization, Cell Morphology, Cellular Function and Maintenance** |

**Table S7**

| **Table S8** | **Protein expression**  % of freshly isolated cells | | | | |  | **Protein degradation** | |  | | **mRNA expression**  % of freshly isolated cells |
| --- | --- | --- | --- | --- | --- | --- | --- | --- | --- | --- | --- |
| **CYP450** | **Protein expression**  24hrs | **Protein expression**  72hrs | | | **Protein expression**  168hrs |  | **Average half-life by 72 hour trend (hrs)** | **Average half-life by 168 hour trend (hrs)** |  | | **mRNA expression at 72hrs**  (Richert *et al*., 2006) |
| **1A2** | 66.94 | 28.88 | | | 22.08 |  | 48.54 | 89.29 |  | | 7.17 |
| **2A6** | 52.68 | 21.68 | | | 9.06 |  | 42.37 | 76.92 |  | | 4.78 |
| **2B6** | 119.38 | 95.1 | | | 81.22 |  | 2500 | 555.56 |  | | 3.21 |
| **2C8** | 110.8 | 54.44 | | | 12.72 |  | 96.15 | 96.15 |  | | 1.38 |
| **2C9** | 247.56 | 205.00 | | | 138.32 |  | - | - |  | | 33.30 |
| **2C18** | 92.43 | 94.90 | | | 92.52 |  | 500 | 1000 |  | | 277.60 |
| **2C19** | 101.96 | 93.85 | | | 52.72 |  | 714.28 | 200 |  | | 17.89 |
| **2D6** | 89.22 | 60.22 | | | 30.86 |  | 92.59 | 116.28 |  | | 11.92 |
| **2E1** | 113.94 | 67.64 | | | 26.44 |  | 142.85 | 119.05 |  | | 2.99 |
| **2J2** | 106.37 | 105.35 | | | 103.23 |  | 2500 | - |  | | 30.80 |
| **3A4** | 106.78 | 34.48 | | | 74.38 |  | 63.29 | 192.31 |  | | 8.58 |
| **4A11** | 93.52 | 52.46 | | | 9.96 |  | 80.65 | 90.91 |  | | 1.88 |
| **4F2** | 201.00 | 154.69 | | | 66.05 |  | - | - |  | | 7.72 |
| **4F3** | 104.98 | 84.23 | | | 64.95 |  | 277.78 | 250 |  | | 4.67 |
| **4F11** | 112.84 | 88.4 | | | 76.76 |  | 555.56 | 384.62 |  | | 79.38 |
| **4F12** | 116.3 | 97.54 | | | 83.08 |  | - | 625 |  | | 17.00 |
| **7B1** | 101.55 | 84.60 | | | 63.93 |  | 263.18 | 238.10 |  | | 77.32 |
| **27A1** | 64.01 | 44.69 | | | 29.98 |  | 59.52 | 102.04 |  | | 6.18 |
| **51A1** | 167.27 | 168.21 | | | 96.22 |  | - | - |  | | 100 |
| **Transporter** | **Protein expression**  24hrs | **Protein expression**  72hrs | | **Protein expression**  168hrs | |  | **Average half-life by 72 hour trend (hrs)** | **Average half-life by 168 hour trend (hrs)** | |  | **mRNA expression at 72hrs**  (Richert *et al*., 2006) |
|  |  | |  |  | |  |  |  |  | |  |
| **BSEP** | 93.12 | | 70.73 | 67.66 | |  | 128.21 | 217.39 |  | | 6.03 |
| **MDR1** | 113.23 | | 132.66 | 143.25 | |  | - | - |  | | 305.33 |
| **MRP2** | 95.98 | | 97.78 | 90.95 | |  | 1250 | 1000 |  | | 182.66 |
| **MRP3** | 121.02 | | 123.91 | 134.63 | |  | - | - |  | | 162.10 |
| **MRP6** | 154.21 | | 81.57 | 56.16 | |  | 12500 | 227.27 |  | | 40.78 |
| **SLC22A7** | 83.33 | | 60.57 | 57.04 | |  | 89.29 | 161.29 |  | | 5.75 |
| **SLCO1B1** | 85.57 | | 42.50 | 50.26 | |  | 64.1 | 131.58 |  | | 12.27 |
| **SLCO1B3** | 115.59 | | 71.76 | 30.38 | |  | 172.41 | 128,21 |  | | 6.10 |
| **SLC22A1** | 101.26 | | 79.37 | 68.09 | |  | 200 | 250 |  | | 6.23 |

| **Upstream Regulator** | **p-value of overlap** |
| --- | --- |
| HNF1A | <0.001 |
| FECH | 0.008 |
| C/ebp | 0.011 |
| SMARCB1 | 0.011 |
| STAT1 | 0.011 |
| STAT6 | 0.012 |
| FOXA1 | 0.012 |
| TNF | 0.038 |
| Ins1 | 0.041 |
| HNF1B | 0.041 |

**Table S9**

| Greater expression in maintained CYP promoters | Greater expression in down-regulated CYP promoters |
| --- | --- |
| HSF2 | NF-Y |
| MITF | ZEB1 |
| Androgen receptor | FOXO1 |
| COUP-TF2 | TBK-1 |
| ATF6 | myogenin / NF-1 |
| Brachyury |  |
| FXR/RXR-alpha |  |
| Nkx3-1 |  |
| Otx2 |  |
| TBP |  |
| TBX5 |  |

**Table S10**
