# Supplementary material for: Mechanistic evaluation of primary human hepatocyte culture using global proteomic analysis reveals a selective dedifferentiation profile
Source: Arch Toxicol. 2016 Apr 2;91(1):439–52. doi: 10.1007/s00204-016-1694-y (PMC5225178; doi:10.1007/s00204-016-1694-y)
Supplement: Supplementary file 14 — Supplementary material 14 (DOCX 17 kb) [file 204_2016_1694_MOESM14_ESM.docx]

**Supplementary figure legends**

**Figure S1:** Dynamic waves of hepatocyte dedifferentiation. Schematic diagram of the time-dependent changes of specific protein groups during dedifferentiation.

**Figure S2:** Pathway analysis of mitochondrial DEPs **a)** Canonical pathway analysis of mitochondrial proteins Results displayed –Log (P values), pathway included if p<0.05 at any of the assessed timepoints. Intensity of purple corresponds to the significance value; **b)** Upstream regulators of mitochondrial changes predicted to be activated or inhibited by IPA software displayed. Red regulators are up-regulated, green regulators are down-regulated. Upstream regulators were included if Z activation scores (2 ≥ Z-score ≤ -2).

**Figure S3:** PANTHER analysis of biological functions of **a)** DEPs at 168 hours; **b)** Non-DEPs at 168 hours; **c)** most variable proteins; **d)** most stable proteins. Proteins divided by function and represented as a pie chart.

**Figure S4:** Correlation between protein expression at 168 hours and mRNA expression at 72 hours in CYPs which are significantly down-regulated at 168 hours. Gene expression data is derived from previously published literature using similar monolayer culture conditions (Richert et al. 2006). P values calculated using linear correlation analysis.

**Supplementary table legends**

**Table S1:** iTRAQ proteomic analysis. Details of the donors analysed in each iTRAQ run and the number of proteins detected in each separate analysis.

**Table S2:** Significantly differentially expressed proteins at each timepoint (P<0.05). Table includes individual donor raw values from each iTRAQ run relative to the corresponding 0 hour sample. Samples ordered according to p-value. Log fold change and Benjamini-Hochberg score for each protein also shown. All proteins in bold are significant (p<0.05) at 168 hours. Log_2_ fold change highlighted in red (up-regulated) or green (down-regulated), with the intensity of the colour representing the degree of change.

**Table S3:** The most variable proteins. Co-efficient of variance analysis used to assess the variability between proteins. Those with a CV>1.3 at any timepoint are listed with their corresponding CV values at each timepoint.

**Table S4:** Pathway analysis of the most variable proteins. The canonical pathways (p<0.05, Fisher exact T-test) significantly associated the most variable proteins (listed in table S2).

**Table S5:** The most stable proteins. Proteins which had a co-efficient of variance of <30% and a mean relative fold change >0.8 and <1.2 throughout the analysis were described as stable.

**Table S6:** The 10 most up and down-regulated proteins by log_2_ fold change at each timepoint 24, 72 and 168 hours relative to freshly isolated PHH.

**Table S7:** Summary of the groups of pathways most significantly associated with DEPs at each timepoint in the cellular and molecular pathways, toxicity pathways and network categories. For each timepoint and category the top 5 pathways/networks are shown.

**Table S8:** Comparison of proteomic CYP and drug transporter expression profile during dedifferentiation with previously reported gene expression and protein degradation rates. Protein half-life calculated using linear trendline at 72 and 168 hours of culture. Gene expression data is derived from previously published literature using similar monolayer culture conditions (Richert et al. 2006)

**Table S9:** Understanding the selective loss of metabolic competence. Most significantly associated upstream regulators of ADME DEPs (p<0.05) as associated by IPA ingenuity software.

**Table S10:** Predicted transcription factor binding. The transcription factors with enriched predicted binding in the upstream regions of either the differentially expressed or non-differentially expressed Cytochrome P450s. The transcription factors were included if enrichment was ≥4 CYPs from either group and are ordered according to the amount of enrichment. Analysis of LETFs promoter regions shows the transcription factors with predicted binding in the greatest number of LETFs. Transcriptions factors are ordered according to the number LETFs with predicted binding sites.

Richert L, Liguori MJ, Abadie C, et al. (2006) Gene expression in human hepatocytes in suspension after isolation is similar to the liver of origin, is not affected by hepatocyte cold storage and cryopreservation, but is strongly changed after hepatocyte plating. Drug Metabolism and Disposition 34(5):870-879 doi:10.1124/dmd.105.007708
